# Comparison of Sensitivity and Specificity of Biparametric versus Multiparametric Prostate MRI in the Detection of Prostate Cancer in 431 Men with Elevated Prostate-Specific Antigen Levels

**DOI:** 10.3390/diagnostics11071223

**Published:** 2021-07-07

**Authors:** Filippo Pesapane, Marzia Acquasanta, Rosario Di Meo, Giorgio Maria Agazzi, Priyan Tantrige, Marina Codari, Simone Schiaffino, Francesca Patella, Anastasia Esseridou, Francesco Sardanelli

**Affiliations:** 1 Department of Biomedical Sciences for Health, Università degli Studi di Milano, Via Santa Sofia, 9, 20122 Milano, Italy; filippo.pesapane@ieo.it (F.P.); francesco.sardanelli@unimi.it (F.S.); 2 Breast Imaging Division, IEO European Institute of Oncology IRCCS, 20141 Milano, Italy; 3 Postgraduation School in Radiodiagnostics, Università degli Studi di Milano, Via Santa Sofia, 9, 20122 Milano, Italy; marzia.acquasanta@unimi.it; 4 Radiology Department, Università degli Studi di Brescia, Brescia, Contrada Santa Chiara, 50, 25122 Brescia, Italy; giorgiomaria.agazzi@gmail.com; 5 Interventional Radiology, King’s College Hospital, Denmark Hill, London SE5 9RS, UK; priyan.tantrige@gmail.com; 6 Dipartimento di Elettronica, Informazione e Bioingegneria, Politecnico di Milano, 20133 Milano, Italy; marina.codari@gmail.com; 7 Radiology Unit, IRCCS Policlinico San Donato, Piazza Edmondo Malan, 2, 20097 San Donato Milanese, Italy; schiaffino.simone@gmail.com (S.S.); bountydoc@libero.it (A.E.); 8 Diagnostic and Interventional Radiology Department, San Paolo Hospital, University of Milan, Via Festa del Perdono, 7, 20122 Milano, Italy; francesca.patella@asst-santipaolocarlo.it

**Keywords:** prostatic neoplasms, magnetic resonance imaging, radiology, sensitivity and specificity

## Abstract

(1) Background: the study of dynamic contrast enhancement (DCE) has a limited role in the detection of prostate cancer (PCa), and there is a growing interest in performing unenhanced biparametric prostate-MRI (bpMRI) instead of the conventional multiparametric-MRI (mpMRI). In this study, we aimed to retrospectively compare the performance of the mpMRI, which includes DCE study, and the unenhanced bpMRI, composed of only T2-weighted imaging and diffusion-weighted imaging (DWI), in PCa detection in men with elevated prostate-specific-antigen (PSA) levels. (2) Methods: a 1.5 T MRI, with an endorectal-coil, was performed on 431 men (aged 61.5 ± 8.3 years) with a PSA ≥4.0 ng/mL. The bpMRI and mpMRI tests were independently assessed in separate sessions by two readers with 5 (R1) and 3 (R2) years of experience. The histopathology or ≥2 years follow-up served as a reference standard. The sensitivity and specificity were calculated with their 95% CI, and McNemar’s and Cohen’s κ statistics were used. (3) Results: in 195/431 (45%) of histopathologically proven PCa cases, 62/195 (32%) were high-grade PCa (GS ≥ 7b) and 133/195 (68%) were low-grade PCa (GS ≤ 7a). The PCa could be excluded by histopathology in 58/431 (14%) and by follow-up in 178/431 (41%) of patients. For bpMRI, the sensitivity was 164/195 (84%, 95% CI: 79–89%) for R1 and 156/195 (80%, 95% CI: 74–86%) for R2; while specificity was 182/236 (77%, 95% CI: 72–82%) for R1 and 175/236 (74%, 95% CI: 68–80%) for R2. For mpMRI, sensitivity was 168/195 (86%, 95% CI: 81–91%) for R1 and 160/195 (82%, 95% CI: 77–87%) for R2; while specificity was 184/236 (78%, 95% CI: 73–83%) for R1 and 177/236 (75%, 95% CI: 69–81%) for R2. Interobserver agreement was substantial for both bpMRI (κ = 0.802) and mpMRI (κ = 0.787). (4) Conclusions: the diagnostic performance of bpMRI and mpMRI were similar, and no high-grade PCa was missed with bpMRI.

## 1. Introduction

Prostate cancer (PCa) is the second leading cause of cancer death in men, and its incidence is expected to double in 10 years. Although the prostate-specific antigen (PSA) is a widely used biomarker, its use is limited by a low specificity and positive predictive value (PPV) for PCa [1], and so its screening use is controversial [2]. In the last decades, multiparametric MRI (mpMRI), including T2-weighted imaging (T2WI), diffusion-weighted imaging (DWI), and dynamic T1-weighted contrast enhancement (DCE) study, became the standard for detecting PCa and improving the biopsy yield by targeting suspicious lesions, while minimizing the risk of unnecessary diagnosis of clinically insignificant PCas [3,4].

However, the safety profile of gadolinium-based contrast agents (GBCA) was recently discussed, including deposition in the brain and other tissues, even in individuals with normal kidney function [5], with an emphasis on repeated injections. Moreover, prostate mpMRI is expensive and time-consuming, and difficult to implement on a large scale as the demand for prostate MRI rises, while MRI capacity remains limited [6]. Over time, it has become evident that DCE sequences rarely significantly alter the MRI diagnosis [6,7,8,9,10]. According to the second version of the prostate imaging reporting and data system (PI-RADS v2) [11], DCE makes a valuable contribution towards risk stratification only in peripheral zone (PZ) lesions and only when the DWI is inconclusive, while it does not have an acknowledged role in the evaluation of the transition zone (TZ).

In recent years, unenhanced MRI, also called biparametric MRI (bpMRI) because it includes only T2WI and DWI, has been proposed as an alternative to mpMRI [6,7,8,9,12]. Omission of the DCE sequences simplifies and shortens the examination and eliminates the costs and risks associated with GBCA administration [6,7,9]. Nevertheless, mpMRI is still the standard practice, with DCE sequences performed as a safety-net, especially when either T2WI and DWI are of inadequate quality, more commonly in older 1.5 T scanners, when the endorectal coil (ERC) is not used [10]. Recently, the PI-RADS Steering Committee updated the PI-RADS guideline with a 2.1 version [10], expressing acknowledgement of the potential benefits and supporting the research into the performance of bpMRI in various clinical scenarios.

The primary objective of the present retrospective study was to determine the diagnostic accuracy of PI-RADS v2 applied to bpMRI in comparison with PI-RADS v2 applied to conventional mpMRI in a cohort of 431 consecutive patients with elevated PSA level (PSA ≥ 4.0 ng/mL) who underwent prostate MRI with ERC at our center. Our secondary objectives were to investigate high-grade and significant PCa detection rates of bpMRI versus mpMRI and interobserver agreement for the interpretation of mpMRI.

## 2. Materials and Methods

### 2.1. Study Design and Patients

This retrospective study was approved by the institutional review board (approval code: 152/int/2019) and it was conducted at a single academic center in a consecutive series of patients. We reviewed prostate MRI examinations performed using 1.5 T systems between January 2010 and December 2016.

Patients who met the following inclusion criteria were selected: (a) mpMRI performed with ERC; and (b) available reference standard in the form of histopathological analysis after fusion MRI-targeted biopsies or radical prostatectomy (RP), in the case of suspected PCa at mpMRI, or a ≥2 years imaging follow-up (MRI or TRUS), in the case of benign results at mpMRI.

In order to correctly apply the PI-RADS v2 [11], we used the following exclusion criteria: (a) prior prostate interventions and therapies; (b) incomplete MRI acquisition, and the presence of imaging artifacts or hemorrhage from prior biopsy; and (c) MRI performed without ERC.

In our population, the score, size, and location of each suspicious lesion were established by a single radiologist according to PI-RADS version 2. Patients with lesions scores ≥3 underwent targeted and systematic biopsies or RP.

Target biopsies were performed by urologists with a fusion MRI-US system, using a commercially available device (UroNav, Invivo, Philips Healthcare, Gainesville, FL, USA), obtaining 6 to 12-core biopsy cores via an 18-gauge automatic spring-loaded biopsy needle. Targeted biopsies were always carried out first, with a maximum of four lesions targeted. Non-targeted biopsies involved sampling nonsuspicious areas on an MRI, although a full transperineal mapping template was not used.

A Gleason score (GS) of ≥ 7 and the presence of extra-prostatic invasion defined the presence of clinically significant or high-grade PCa (namely Gleason grade group > 3 and ISUP grade group > 3) [13,14].

### 2.2. MRI Technique

The acquisition parameters used for the study are presented in Table 1.

All prostate MRI examinations were performed with the patient in the supine position using a 1.5 T unit. From 2010 to 2014, the unit used was a Magnetom Sonata, while from 2014 to 2016 it was a Magnetom Symphony (Siemens Healthineers). In both cases, they were equipped with 40 mT/m gradients using a 4-channel surface coil (Body Matrix, A Tim Coil, Siemens Healthineers) and a single-channel ERC (Medrad eCoil, MR Endorectal Coil, Bayer Healthcare, Berlin, Germany).

The mpMRI protocol included: an axial T1-weighted sequence with fat saturation; a three-plane T2WI sequence; an axial echo-planar DWI sequence; and a three-dimensional T1-weighted DCE study.

From 2010 to 2011, gadobenate dimeglumine (MultiHance, Bracco) was used in a dose of 0.1 mM/kg of body weight, immediately followed by 20 mL of saline flush (flow rate of 2 mL/s); whereas, from 2012 to 2016, gadoteridol (Prohance, Bracco) was used in a dose of 0.1 mM/kg of body weight, immediately followed by 20 mL of saline flush (flow rate of 2 mL/s).

The average of the total examination time (including the cannulation and injection of contrast, and the positioning of ERC) for each examination was approximately 45 min.

### 2.3. Image Analysis

All retrieved images were anonymized and independently read by two radiologists, who were blinded to the clinical findings, histopathology results, and follow-up. Reader 1 (R1) had 5 years of experience and reader 2 (R2) had 3 years of experience (with around 500 prostate MRIs conducted in a year) in the interpretation of prostate MRIs.

Initially, all MRI datasets were interpreted and classified according to PI-RADS v2 [11], with a biparametric approach, i.e., including only three-plane (axial, sagittal, and coronal) T2WI and axial DWI with corresponding maps of the apparent diffusion coefficient (ADC), without any evaluation of the DCE study. The index lesion was considered as the lesion with the highest PI-RADS v2 score. When more than one lesion with the same PI-RADS v2 score was detected, the larger lesion was considered as the index lesion. Any discrepancies between the two radiologists about the localization of the index lesion were resolved through discussion to reach a consensus. Using the biparametric approach, lesions with DWI scores of 3 were directly classified as PI-RADS 3 without further evaluation with DCE sequences (which were omitted by definition).

As a second step, in a different reading session (with a time gap of more than one month), the DCE sequences were also included and each mpMRI examination was evaluated as a new exam, according to PI-RADS v2 [11].

Hence, the results of mpMRI were individually compared against the previous bpMRI results, to correlate the PI-RADS v2 scores. An overall assessment score of 3 was used as a threshold for a test-positive result (for both bpMRI and mpMRI). Accordingly, the categories of 1 or 2 for the index lesion were considered a test-negative result, while a category of 3, 4, or 5 were considered a test-positive result.

### 2.4. Statistical Analysis

Using dichotomized results (with an overall assessment score of 3 as the threshold for a positive test), sensitivity, specificity, PPV, and negative predictive values (NPV), alongside the overall diagnostic accuracy of the bpMRI, were analyzed and compared to that of the mpMRI for the diagnosis of PCa by using McNemar’s test and 95% Clopper-Pearson confidence intervals (CIs), which were established for all proportions.

All analyses were two-tailed and a *p* value of less than 0.05 was considered as significant.

Inter-observer reproducibility between the two readers was calculated for the screen-reading strategy using Cohen κ statistics. The κ values were considered as follows: 0–0.20, slight agreement; 0.21–0.40, fair agreement; 0.41–0.60, moderate agreement; 0.61–0.80, substantial agreement; and 0.81–1 almost perfect agreement [15].

Statistical calculations were performed using SPSS Statistics (IBM Corp. Released 2017. IBM SPSS Statistics, V25.0. IBM, Armonk, NY, USA).

## 3. Results

**Figure 1:**
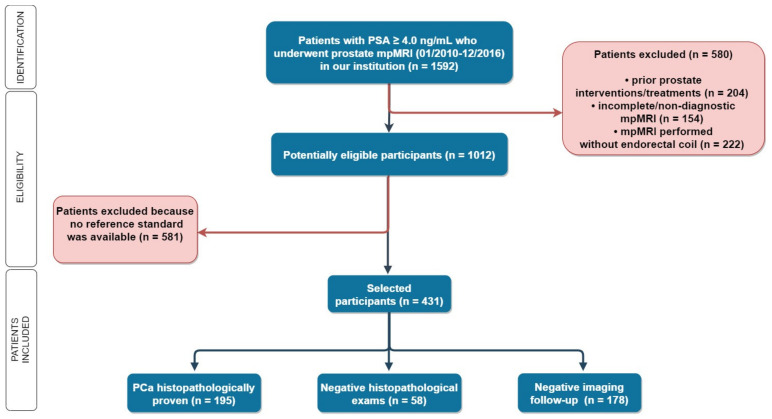
Flowchart: 1592 patients with elevated PSA levels (≥4.0 ng/mL) underwent mpMRI in our institution, and 431 patients were selected according to our inclusion and exclusion criteria. In 253 patients, a histopathological reference standard was available. PSA: Prostate-specific antigen, mpMRI: multiparametric MRI, and PCa: prostate cancer.

Out of 1592 men with elevated PSA levels (≥ 4.0 ng/mL) who underwent mpMRI during the study period, 431 met the inclusion criteria (Figure 1 and Table 2).

Given a pretest probability of 43%, the positive post-test probability was 85%, and the negative post-test probability was 18%. The histopathological reference standard was available for 253 patients (namely, 151 RP, 65 systematic biopsies, and 37 targeted biopsies), of whom 195/431 (45%) were found to have histopathologically proven PCa, originating in the PZ (*n* = 134, 69%) or transitional zone (TZ) (*n* = 61, 31%). High-grade PCa (namely, Gleason grade group ≥ 2/ISUP grade group ≥ 2) [13,14] was identified in 65/195 (33%), while low-grade PCa, defined as GS ≤ 6 (namely, Gleason grade group = 1/ISUP grade group = 1) [13,14] was present in 130/195 (67%). PCa was excluded by histopathology in 58/431 (14%) patients and by ≥2 years follow-up in 178/431 (41%) patients: among these patients, during the 2-year follow-up, 31/236 (13%) had a false negative initially.

Table 3 shows the diagnostic performance of R1 and R2 for both mpMRI and bpMRI in the detection of any PCa and in the detection of high-grade PCa.

Overall, 12 discrepancies between R1 and R2 about the localization of the index lesion reached a consensus through discussion.

**Figure 2:**
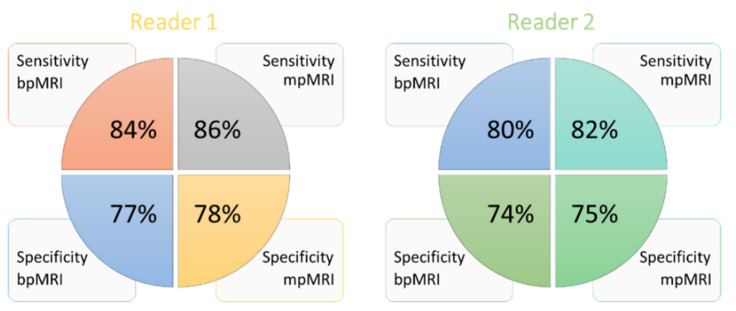
Sensitivity and specificity performance of each reader in bpMRI and mpMRI. Reader 1 had a sensitivity and specificity of 84% and 77% for bpMRI, respectively, versus 86% and 78% for mpMRI. Reader 2 had a sensitivity and specificity of 80% and 74% for bpMRI, respectively versus 82% and 75% for mpMRI. bpMRI: biparametric MRI, and mpMRI: multiparametric MRI.

Considering all PCa cases (with any GS), for bpMRI, the sensitivity was 84% and 80%, and the specificity was 77% and 74% for R1 and R2, respectively; while for mpMRI, the sensitivity was 86% and 82%, and the specificity was 78% and 75%, for R1 and R2, respectively (Figure 2).

Considering high-grade PCa cases, for bpMRI, R1 and R2 had a sensitivity of 84% and 80%, and a specificity of 77% and 74%, respectively; while, for mpMRI, sensitivity was 86% and 80%, and specificity was 78% and 74%, respectively.

Overall, we reported 82% sensitivity, 75.5% specificity, and 78.5% accuracy for bpMRI, and 84% sensitivity, 76.5% specificity, and 80% accuracy for mpMRI. Considering high-grade PCa only, we reported 82% sensitivity, 75.5% specificity, and 76.5% accuracy for bpMRI, while 83% sensitivity, 76% specificity, and 77.5% accuracy was reported for mpMRI.

Table 4 shows the distribution of mpMRI and bpMRI index lesions for PI-RADS v2 scores according to each of the two readers. When comparing bpMRI with mpMRI, the number of patients scored identically were 406/431 (94.2%) and 396/431 (91.9%) by R1 and R2, respectively, with an adequate distribution of diagnostic scores and with relatively numerous scores of 2, allowing for a reliable evaluation of specificity. Accordingly, evaluation of bpMRI alone led to a change in the score, when compared to mpMRI, in 25/431 (6%) of cases for R1 and in 35/431 (8%) of cases for R2.

Patients with PI-RADS 1 were underrepresented in this study, as they usually received neither a histologic workup nor an imaging follow-up MRI. The main consequence of omitting DCE was an increased number of lesions scored 3: the frequency of PI-RADS score 3 increased by 24/431 (6%) cases for R1 and by 32/431 (7%) cases for R2. This was due to the structure of PI-RADS v2, where DCE was mainly used to further investigate inconclusive DWI results of an index lesion in the PZ, thus we can upgrade PI-RADS 3 to PI-RADS 4 [11].

The biparametric MRI resulted in 4 more false negative cases, compared to mpMRI, for both R1 and R2. Of note, these 4 false negative cases in the bpMRI cohort (correctly identified using mpMRI) were low-grade PCa (GS ≤ 6). Both readers assessed as negative the same two patients with GS = 3 + 3 and GS = 3 + 4, respectively. The other 4 false negative cases were GS = 3 + 4 and GS = 2 + 4 for R1, and GS = 3 + 3 and GS = 3 + 4 for R2. No high-grade PCa (GS ≥ 7) was missed with bpMRI.

No significant differences in sensitivity and specificity were observed in both bpMRI and mpMRI by either R1 (*p* = 0.092 and *p* = 0.096, respectively) or R2 (*p* = 0.117 and *p* = 0.133, respectively).

There was no significant difference reported between the sensitivity and specificity of the mpMRI with the two different contrast agents used in the two study periods (*p* = 0.182 and *p* = 0.106, respectively).

Cohen’s kappa was substantial for both bpMRI (κ = 0.802) and mpMRI (κ = 0.787) in the evaluation of interobserver agreement across the two readers.

**Figure 3:**
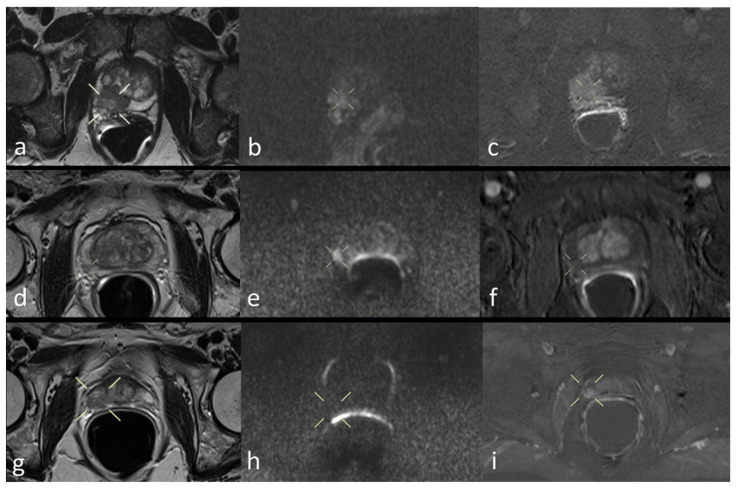
Prostate mpMRI of three patients: T2WI (**a**), DWI (**b**), and DCE image (**c**) in a true positive patient; T2WI (**d**), DWI (**e**), and DCE image (**f**) in a false positive patient; T2WI (**g**), DWI (**h**), and DCE image (**i**) in a false negative patient.

Finally, Figure 3 shows three patient study images of T2, DWI, and perfusion as a proof of quality.

## 4. Discussion

In our study, when PI-RADS v2 was applied to an unenhanced prostate bpMRI, it did not provide significantly different sensitivity and specificity than when applied to mpMRI; with an accuracy of 80% (R1) and 77% (R2), it was consistent with previous results [6,7,8,16,17]. No high-grade PCa was missed by bpMRI. These findings confirmed bpMRI as a potential triage test in men with elevated PSA, avoiding unnecessary biopsies and the inherent complications (e.g., infection and rectal bleeding) [18].

Although the PI-RADS v2.1 guideline indicated DWI and T2WI as dominant sequences for PCa detection in the PZ and TZ, respectively, the necessity of DCE was discussed [16]. Since DCE has some drawbacks, its omission would simplify the examination and eliminate the cost and potential GBCA-associated problems, including concerns for tissue gadolinium retention [5,19,20].

High accuracy of unenhanced prostate MRI, combined with PSA evaluation, was shown for the detection of clinically significant PCa [8,12,21,22], and a meta-analysis showed that bpMRI, with a high b-value, is a sensitive tool for diagnosing PCa [23]. We considered previous studies, both those in favor of the added value of DCE [24,25] and those against it [21,26], arguing that different results may have been caused by heterogeneity in technical issues, study populations, and interpretation criteria. Thus, for the sake reproducibility, we used the PI-RADS v2 scoring system, designed for mpMRI including DCE, and included all patients examined in the study period who had increased PSA in compliance with the PI-RADS v2 criteria and appropriate image quality via the use of ERC.

Our results showed no significant differences in sensitivity and specificity between bpMRI and mpMRI, at 80–84% and 70–74%, respectively, which was consistent with two recent large meta-analyses based on 2383 patients (sensitivity: 76–85%; specificity: 69–84%) [16] and 627 (sensitivity: 66–81%; specificity: 86–93%) [27] patients. Moreover, recent single-center studies [6,8,17] matched our findings. Noteworthy, Barth et al. [28], through looking only at axial images, showed that two sequences alone (axial T2WI and axial DWI) performed equally as well as mpMRI including coronal and sagittal images. In addition, we also had substantial interobserver agreement.

Although DCE may allow for the identification of subtle lesions missed using unenhanced sequences [16], De Viscchere et al. [7] showed that in 19.2% of patients in whom the DCE findings were needed for the determination of the overall assessment category, the supplementary information was incorrect in approximately 30%. Similarly, Greer et al. [29] demonstrated that a high false positive rate (129/204, 63.2%) may be decreased by avoiding DCE. In fact, they may be misleading due to the overlapping between benign prostate hyperplasia and possible false negative low-grade PCas [8]. This may explain why, in our study, the readers’ agreement for mpMRI (κ = 0.787) was slightly lower than for bpMRI (κ = 0.802), although both were substantial.

In addition to the main advantage of avoiding GBCA administration and the related safety problem of its tissue deposition, bpMRI reduces costs and shortens examination times. Our choice to consider the use of ERC as an inclusion criterion would seem at cross-purposes, as the ERC placement is time-consuming. On the contrary, the use of ERC made our study a unique demonstration in literature, in that bpMRI may be clinically usable with a 1.5 T MR: as our selected MRI exams, performed with ERC, fulfilled the quality criteria for inclusion, we showed that a bpMRI may not need a 3 T MR to reach similar performances as a mpMRI.

Our study has limitations. First, this was a retrospective single-center study, and prospective studies are still needed to prove whether bpMRI is equal to a mpMRI in the diagnosis of clinically significant PCa. Second, our study population was not homogeneous and step-section histopathology of RP specimens was not performed. However, such a population better represents real-world clinical practice as studies using only RP specimens as the reference standard may show selection bias via the exclusion of men with negative biopsy or patients not suitable for RP. Third, the standard of reference was not determined by a per-lesion basis, but only on a per-patient basis, without histological confirmation of PCa-absence in the cases with negative follow-up, and with the chance of missing PCa in both bpMRI and mpMRI. Finally, we did not investigate the bpMRI value for other clinical applications such as PCa staging, for which DCE may still play a required role, because it was beyond our purposes.

## 5. Conclusions

In our population, the retrospective application of PI-RADS v2 to the bpMRI of 431 men with increased PSA showed no significant reduction in diagnostic performance, as compared to its application to mpMRI, and without missing any high-grade cancer. Accordingly, bpMRI may play a role as a triage test in men with elevated PSA to improve risk stratification and exclude aggressive PCa.

## Figures and Tables

**Table 1:** MRI Parameters: mpMRI acquisition parameters. Differences in parameters between two scans, noted with *, were considered negligible.

	Magnetom Sonata, A Tim System 1.5 T eco, Siemens Healthineers, Germany	Magnetom Symphony, A Tim System 1.5 T eco, Siemens Healthineers, Germany
T2-weighted imaging	Axial, coronal, sagittal T2 TSE, 3 mm slice thickness, multi-slice mode: interleaved	Axial, coronal, sagittal T2 TSE, 3 mm slice thickness, multi-slice mode: interleaved
	TR 5100 ms, TE 111 ms, FA 150°, FOV 240 mm × 100 mm, base resolution 256 with voxel 1.1 mm × 0.9 mm × 3.0 mm, number of averages 1	TR 5540 ms, TE 101 ms, FA 150°, FOV 240 mm × 100 mm, base resolution 256 with voxel 1.1 mm× 0.9 mm × 3.0 mm, number of averages 1
Diffusion weighted imaging	Transverse REVEAL, 3 mm slice thickness	Transverse REVEAL, 3 mm slice thickness
	b-values 0, 700, and 1400 (calculated)	b-values 0, 700, and 1400 (calculated)
	TR 5100 ms *, TE 90 ms *, number of averages: 2	TR 6000 ms *, TE 111 ms *, number of averages: 2
Dynamic contrast enhanced imaging	Transverse T1-VIBE (fat saturated)	Transverse T1-VIBE (fat saturated)
	3 mm slice thickness	3 mm slice thickness
	Perfusion temporal resolution: 15 s (total time length: 360 s) TR 8.8 ms *, TE 3.88 ms, FA 14°	Perfusion temporal resolution: 15 s (total time length: 360 s) TR 8.7 ms *, TE 3.88 ms, FA 14°

TSE: turbo spin echo, TE: echo time, FA: flip angle, FOV: field of view, mm: millimeters, TR: repetition time, and ms: milliseconds.

**Table 2:** Patient characteristics: characteristics of the 431 patients selected in the study.

PATIENT CHARACTERISTICS	MEAN/MEDIAN (RANGE)	SD/IQR
**AGE**	61.5 (49–84) years	8.3 (SD)
**PROSTATE VOLUME**	58 (25–108) mL	19–201 (IQR)
**PSA**	12.0 (4.4–90) ng/mL	4.4–87.2 (IQR)
**FREE PSA**	19%	8.2–52.4 (IQR)
**PSA DENSITY**	0.18 ng/mL^2^	0.09–0.39 (IQR)

IQR: interquartile range, SD: standard deviation, cm: centimeter, ng: nanogram, and mL: milliliter. PSA: Prostate-specific antigen.

**Table 3:** Diagnostic performance of each reader for both mpMRI and bpMRI.

**bpMRI**												
	**Detection of PCa (Any GS)**						**Detection of High-Grade PCa (GS > 7)**					
	**Reader 1**			**Reader 2**			**Reader 1**			**Reader 2**		
		**95 % CI**			**95 % CI**			**95 % CI**			**95 % CI**	
**Sens**	0.84	0.79	0.89	0.80	0.74	0.86	0.84	0.75	0.93	0.80	0.70	0.90
**Spec**	0.77	0.72	0.82	0.74	0.68	0.80	0.77	0.73	0.81	0.74	0.70	0.78
**PPV**	0.75	0.69	0.81	0.72	0.66	0.78	0.39	0.31	0.47	0.35	0.28	0.43
**NPV**	0.85	0.81	0.90	0.82	0.77	0.87	0.96	0.94	0.99	0.95	0.93	0.98
**1-NPV**	0.15	0.10	0.19	0.18	0.13	0.23	0.04	0.01	0.06	0.05	0.02	0.07
**LR+**	3.65	2.87	4.65	3.08	2.45	3.86	3.65	2.94	4.53	3.08	2.49	3.80
**LR−**	0.21	0.15	3.82	0.27	0.20	3.21	0.21	0.12	1.71	0.27	0.17	1.52
**DOR**	17.58	10.79	28.64	11.38	7.22	17.96	17.58	8.67	35.62	11.38	5.94	21.83
**Accuracy**	0.80			0.77			0.78			0.75		
**mpMRI**												
	**Detection of PCa (Any GS)**						**Detection of High-Grade PCa (GS > 7)**					
	**Reader 1**			**Reader 2**			**Reader 1**			**Reader 2**		
		**95 % CI**			**95 % CI**			**95 % CI**			**95 % CI**	
**Sens**	0.86	0.81	0.91	0.82	0.77	0.87	0.86	0.78	0.94	0.80	0.70	0.90
**Spec**	0.78	0.73	0.83	0.75	0.69	0.81	0.78	0.74	0.82	0.74	0.70	0.78
**PPV**	0.76	0.71	0.82	0.73	0.67	0.79	0.41	0.33	0.49	0.35	0.28	0.43
**NPV**	0.87	0.83	0.92	0.83	0.78	0.88	0.97	0.95	0.99	0.95	0.93	0.98
**1-NPV**	0.13	0.08	0.17	0.17	0.12	0.22	0.03	0.01	0.05	0.05	0.02	0.07
**LR+**	3.91	3.05	5.00	3.28	2.60	4.13	3.91	3.15	4.85	3.08	2.49	3.80
**LR−**	0.18	0.13	4.10	0.24	0.18	3.43	0.18	0.10	1.81	0.27	0.17	1.52
**DOR**	21.78	13.10	36.21	13.67	8.55	21.85	21.78	10.36	45.78	11.38	5.94	21.83
**Accuracy**	0.82			0.78			0.79			0.76		

PCa: Prostate Cancer, GS: Gleason Score, Sens: sensitivity, Spec: specificity, PPV: positive predictive value, NPV: negative predictive value, LR: likelihood ratio, and DOR: diagnostic odds ratio.

**Table 4:** Distribution of PI-RADS scores: distribution of mpMRI and bpMRI index lesions (*n*, %) on the different PI-RADS score levels of the two readers.

**Reader 1**						
	**mpMRI**		**bpMRI**		**Difference mpMRI vs. bpMRI**	
**PI-RADS 2.0 Score**	***n***	**%**	***n***	**%**	***n***	**%**
1	4	0.9	3	0.7	−1	0.4
2	209	48.5	210	48.7	+1	0.4
3	95	22.3	119	27.6	+24	5.6
4	87	20.4	63	14.6	−24	5.6
5	36	8.3	36	8.3	0	0
**Reader 2**						
	**mpMRI**		**bpMRI**		**Difference +/−**	
**PI-RADS 2.0 Score**	*n*	**%**	***n***	**%**	***n***	**%**
1	15	3.5	14	3.2	−1	0.2
2	197	45.7	200	46.4	+3	0.7
3	100	23.2	132	30.6	+32	7.4
4	81	18.8	49	11.4	−32	7.4
5	38	8.8	36	8.4	−2	0.5

mpMRI: multiparametric MRI, and bpMRI: biparametric MRI.

## Data Availability

The data presented in this study are available on request from the corresponding author. The data are not publicly available due to our Institute policy.

## Abbreviations

bpMRI	biparametric MRI
mpMRI	multiparametric MRI
ERC	endorectal coil
GBCA	gadolinium-based contrast agent
GS	Gleason score
PCa	prostate cancer
PZ	peripheral zone
RP	radical prostatectomy
TZ	transition zone

## References

[1] Catalona W.J., Southwick P.C., Slawin K.M., Partin A.W., Brawer M.K., Flanigan R.C., Patel A., Richie J.P., Walsh P.C., Scardino P.T. (2000). Comparison of percent free PSA, PSA density, and age-specific PSA cutoffs for prostate cancer detection and staging. Urology.

[2] Eggener S.E., Cifu A.S., Nabhan C. (2015). Prostate Cancer Screening. JAMA.

[3] Ahmed H.U., Bosaily A.E.S., Brown L.C., Gabe R., Kaplan R., Parmar M.K., Collaco-Moraes Y., Ward K., Hindley R.G., Freeman A. (2017). Diagnostic accuracy of multi-parametric MRI and TRUS biopsy in prostate cancer (PROMIS): A paired validating confirmatory study. Lancet.

[4] Barentsz J.O., Richenberg J., Clements R., Choyke P., Verma S., Villeirs G., Rouviere O., Logager V., Futterer J.J. (2012). European Society of Urogenital, R. ESUR prostate MR guidelines 2012. Eur. Radiol..

[5] McDonald R.J., McDonald J.S., Kallmes D.F., Jentoft M.E., Paolini M.A., Murray D.L., Williamson E.E., Eckel L.J. (2017). Gadolinium Deposition in Human Brain Tissues after Contrast-enhanced MR Imaging in Adult Patients without Intracranial Abnormalities. Radiology.

[6] Junker D., Steinkohl F., Fritz V., Bektic J., Tokas T., Aigner F., Herrmann T.R.W., Rieger M., Nagele U. (2018). Comparison of multiparametric and biparametric MRI of the prostate: Are gadolinium-based contrast agents needed for routine examinations?. World J. Urol..

[7] De Visschere P., Lumen N., Ost P., Decaestecker K., Pattyn E., Villeirs G. (2017). Dynamic contrast-enhanced imaging has limited added value over T2-weighted imaging and diffusion-weighted imaging when using PI-RADSv2 for diagnosis of clinically significant prostate cancer in patients with elevated PSA. Clin. Radio. L..

[8] Di Campli E., Delli Pizzi A., Seccia B., Cianci R., d’Annibale M., Colasante A., Cinalli S., Castellan P., Navarra R., Iantorno R. (2018). Diagnostic accuracy of biparametric vs multiparametric MRI in clinically significant prostate cancer: Comparison between readers with different experience. Eur. J. Radiol..

[9] Stanzione A., Imbriaco M., Cocozza S., Fusco F., Rusconi G., Nappi C., Mirone V., Mangiapia F., Brunetti A., Ragozzino A. (2016). Biparametric 3T Magnetic Resonance Imaging for prostatic cancer detection in a biopsy-naive patient population: A further improvement of PI-RADS v2?. Eur. J. Radiol..

[10] Turkbey B., Rosenkrantz A.B., Haider M.A., Padhani A.R., Villeirs G., Macura K.J., Tempany C.M., Choyke P.L., Cornud F., Margolis D.J. (2019). Prostate Imaging Reporting and Data System Version 2.1: 2019 Update of Prostate Imaging Reporting and Data System Version 2. Eur. Urol..

[11] Weinreb J.C., Barentsz J.O., Choyke P.L., Cornud F., Haider M.A., Macura K.J., Margolis D., Schnall M.D., Shtern F., Tempany C.M. (2016). PI-RADS Prostate Imaging-Reporting and Data System: 2015, Version 2. Eur. Urol..

[12] Rais-Bahrami S., Siddiqui M.M., Vourganti S., Turkbey B., Rastinehad A.R., Stamatakis L., Truong H., Walton-Diaz A., Hoang A.N., Nix J.W. (2015). Diagnostic value of biparametric magnetic resonance imaging (MRI) as an adjunct to prostate-specific antigen (PSA)-based detection of prostate cancer in men without prior biopsies. BJU Int..

[13] Gordetsky J., Epstein J. (2016). Grading of prostatic adenocarcinoma: Current state and prognostic implications. Diagn. Pathol..

[14] Samaratunga H., Montironi R., True L., Epstein J.I., Griffiths D.F., Humphrey P.A., van der Kwast T., Wheeler T.M., Srigley J.R., Delahunt B. (2011). International Society of Urological Pathology (ISUP) Consensus Conference on Handling and Staging of Radical Prostatectomy Specimens. Working group 1: Specimen handling. Mod. Pathol..

[15] Landis J.R., Koch G.G. (1977). The measurement of observer agreement for categorical data. Biometrics.

[16] Niu X.K., Chen X.H., Chen Z.F., Chen L., Li J., Peng T. (2018). Diagnostic Performance of Biparametric MRI for Detection of Prostate Cancer: A Systematic Review and Meta-Analysis. Am. J. Roentgenol..

[17] Choi M.H., Lee Y.J., Jung S.E., Rha S.E., Byun J.Y. (2018). Prebiopsy biparametric MRI: Differences of PI-RADS version 2 in patients with different PSA levels. Clin. Radiol..

[18] Liss M.A., Ehdaie B., Loeb S., Meng M.V., Raman J.D., Spears V., Stroup S.P. (2017). An Update of the American Urological Association White Paper on the Prevention and Treatment of the More Common Complications Related to Prostate Biopsy. J. Urol..

[19] Wagner B., Drel V., Gorin Y. (2016). Pathophysiology of gadolinium-associated systemic fibrosis. Am. J. Physiol. Ren. Physiol..

[20] Jung J.W., Kang H.R., Kim M.H., Lee W., Min K.U., Han M.H., Cho S.H. (2012). Immediate hypersensitivity reaction to gadolinium-based MR contrast media. Radiology.

[21] Delongchamps N.B., Rouanne M., Flam T., Beuvon F., Liberatore M., Zerbib M., Cornud F. (2011). Multiparametric magnetic resonance imaging for the detection and localization of prostate cancer: Combination of T2-weighted, dynamic contrast-enhanced and diffusion-weighted imaging. BJU Int..

[22] Pesapane F., Standaert C., De Visschere P., Villeirs G. (2020). T-staging of prostate cancer: Identification of useful signs to standardize detection of posterolateral extraprostatic extension on prostate MRI. Clin. Imaging.

[23] Liang Z., Hu R., Yang Y., An N., Duo X., Liu Z., Shi S., Liu X. (2020). Is dynamic contrast enhancement still necessary in multiparametric magnetic resonance for diagnosis of prostate cancer: A systematic review and meta-analysis. Transl. Androl. Urol..

[24] Tamada T., Sone T., Higashi H., Jo Y., Yamamoto A., Kanki A., Ito K. (2011). Prostate cancer detection in patients with total serum prostate-specific antigen levels of 4-10 ng/mL: Diagnostic efficacy of diffusion-weighted imaging, dynamic contrast-enhanced MRI, and T2-weighted imaging. Am. J. Roentgenol..

[25] Delongchamps N.B., Beuvon F., Eiss D., Flam T., Muradyan N., Zerbib M., Peyromaure M., Cornud F. (2011). Multiparametric MRI is helpful to predict tumor focality, stage, and size in patients diagnosed with unilateral low-risk prostate cancer. Prostate Cancer Prostatic Dis..

[26] Schimmoller L., Quentin M., Arsov C., Hiester A., Buchbender C., Rabenalt R., Albers P., Antoch G., Blondin D. (2014). MR-sequences for prostate cancer diagnostics: Validation based on the PI-RADS scoring system and targeted MR-guided in-bore biopsy. Eur. Radiol..

[27] Woo S., Suh C.H., Kim S.Y., Cho J.Y., Kim S.H., Moon M.H. (2018). Head-to-Head Comparison Between Biparametric and Multiparametric MRI for the Diagnosis of Prostate Cancer: A Systematic Review and Meta-Analysis. Am. J. Roentgenol..

[28] Barth B.K., De Visschere P.J.L., Cornelius A., Nicolau C., Vargas H.A., Eberli D., Donati O.F. (2017). Detection of Clinically Significant Prostate Cancer: Short Dual-Pulse Sequence versus Standard Multiparametric MR Imaging-A Multireader Study. Radiology.

[29] Greer M.D., Shih J.H., Lay N., Barrett T., Kayat Bittencourt L., Borofsky S., Kabakus I.M., Law Y.M., Marko J., Shebel H. (2017). Validation of the Dominant Sequence Paradigm and Role of Dynamic Contrast-enhanced Imaging in PI-RADS Version 2. Radiology.

